# Choroidal detachment with exudative retinal detachment following Ahmed valve implantation in Sturge-Weber syndrome

**DOI:** 10.3205/oc000122

**Published:** 2019-10-25

**Authors:** Bipul Kumer De Sarker, Ginger Helen, Mohammad Ibn Abdul Malek, Abdullahi Sadiq, Zafrul Hassan, Jahangir Kabir, Sarat Badmus, Iftekhar Sazzad, Mostafizur Rahman, Mallika Mahatma, Abdus Salam

**Affiliations:** 1 Glaucoma, Ispahani Islamia Eye Institute and Hospital, Dhaka, Bangladesh; 2 Retina, Ispahani Islamia Eye Institute and Hospital, Dhaka, Bangladesh; 3 Paediatric and Strabismus, Ispahani Islamia Eye Institute and Hospital, Dhaka, Bangladesh; 4 Pathology and Microbiology, Ispahani Islamia Eye Institute and Hospital, Dhaka, Bangladesh; 5 Cornea, Ispahani Islamia Eye Institute and Hospital, Dhaka, Bangladesh

**Keywords:** Ahmed glaucoma valve, Sturge-Weber syndrome, choroidal hemangioma, choroidal detachment

## Abstract

Ahmed glaucoma valve implant appears to be a relatively useful drainage device in eyes with glaucoma secondary to Sturge-Weber syndrome (SWS). However, early postoperative choroidal and exudative retinal detachment may occur from a rapid expansion of the choroidal hemangioma with effusion of fluid into the suprachoroidal and subretinal spaces. We report the case of a ten-year-old boy who had SWS with choroidal haemangioma and secondary glaucoma. He had Ahmed glaucoma valve implantation on account of the secondary glaucoma which had been refractory to both conventional medical and surgical managements. He developed choroidal and exudative retinal detachment postoperatively. However, he responded to conservative treatment and further surgical management was not required.

Ahmed glaucoma valve in the treatment of glaucoma secondary to SWS is useful, but the risk of choroidal effusion with exudative retinal detachment is still present. Surgeons should be alert to this likely complication and be prepared for prompt management.

## Introduction

Secondary glaucoma from Sturge-Weber syndrome (SWS) occurs when facial haemangioma involves the lids or conjunctiva [1]. The onset of glaucoma may present from infancy to early adulthood. In older children, the elevated IOP is due to an elevation of episcleral venous pressure that occurs as a result of arteriovenous shunts through the episcleral hemangiomas [2].

Choroidal haemangiomas and episcleral haemangiomas are commonly seen, and leakage from the choroidal hemangioma may cause retinal oedema [1]. 

In older children, medical therapy may be better tolerated and effective, with fewer side effects. If medical therapy is unsuccessful, either filtration surgery or tube surgery can be used [1]. However, in 24% of cases with SWS trabeculectomy had intraoperative choroidal detachment from a rapid expansion of the choroidal hemangioma with effusion of fluid into the suprachoroidal and subretinal spaces [3]. Studies have also shown that choroidal detachment occurred in some patients with SWS following Ahmed valve implantation [4], [5].

Oral propranolol has been reported to be useful in treatment of circumscribed choroidal hemangioma [6]. Its use in treating diffuse choroidal haemangioma preoperatively has been suggested to prevent choroidal detachment [6].

## Case description

A 10-year-old male child with SWS presented to the glaucoma department of Ispahani Islamia Eye Institute and Hospital, Bangladesh with raised intraocular pressure (IOP) in the left eye. He had undergone trabeculectomy in the same eye with mitomycin C before presentation. On examination, his best corrected visual acuity was 6/6 on the right eye and 6/9 on the left. His intraocular pressure was 15 mmHg in the right eye and 37 mmHg in the left eye.

Slit-lamp examination of the right eye was within normal limits while that of the left eye showed a flat, avascular superior bleb with a patent superior peripheral iridectomy. The cornea was clear and the peripheral anterior chamber depth was equal to the corneal thickness.

**Figure 1:**
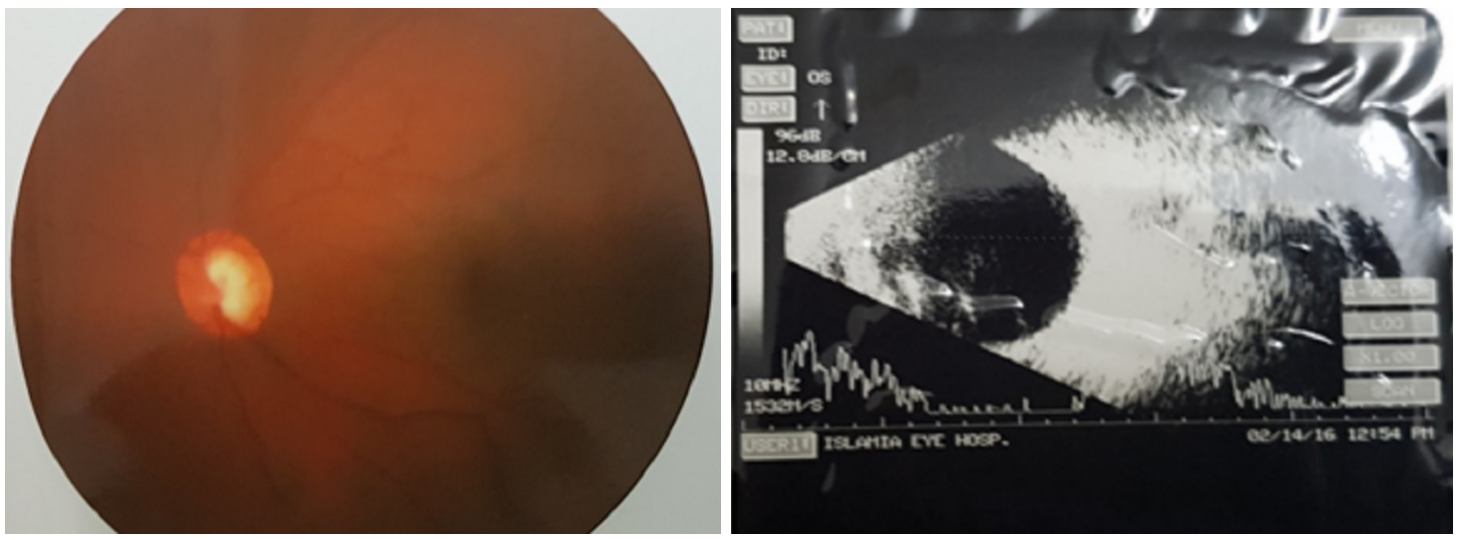
Fundus photograph of the left eye showing a vertical cup disc ratio of 0.7 and B-scan ultrasonography showing no choroidal detachment and no retinal detachment

Fundoscopy revealed a cup disc ratio of 0.7 in the left eye and was documented with a colour fundus photograph (Figure 1 (Fig. 1)). B-scan ultrasonography of the left eye revealed a diffuse choroidal haemangioma with no retinal detachment (Figure 1 (Fig. 1)).

The patient was placed on dorzolamide timolol combination eye drops every eight hours and travoprost 0.004% once daily at night. He also had oral acetazolamide tablets 250 mg twice daily for fifteen days from presentation. Follow-up of this patient revealed a progression of optic nerve damage and a persistently raised intraocular pressure of 23 mmHg on the left eye despite the use of maximal antiglaucoma medication. Therefore, he was planned for and underwent uneventful Ahmed glaucoma valve implantation over the superior temporal quadrant in the left eye under general anaesthesia. Postoperatively, the patient was placed on moxifloxacin eye drop four-hourly, prednisolone eye drop two-hourly, homatropine 2% eye drop eight-hourly and dexamethasone sodium ointment at night. 

**Figure 2:**
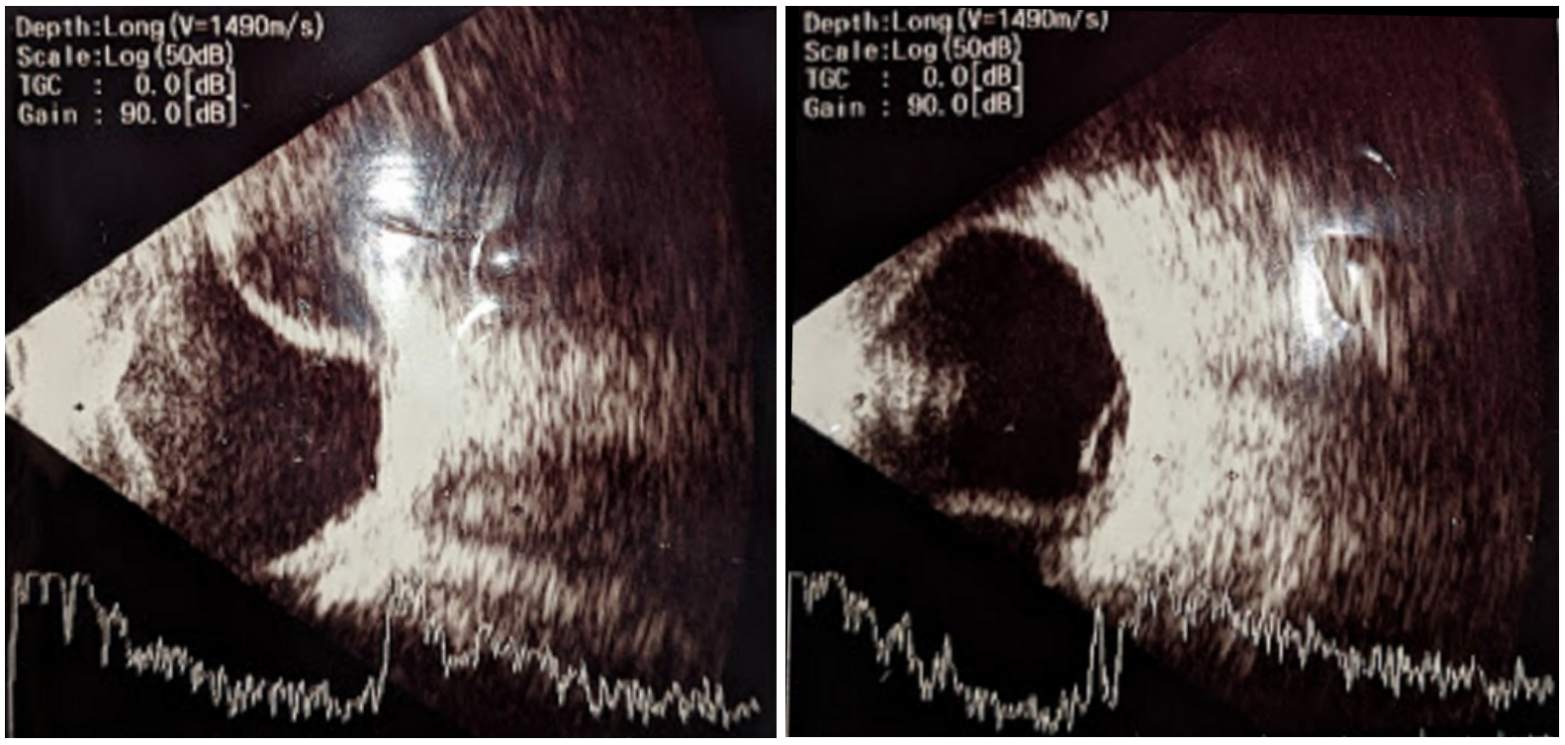
B-scan ultrasonography showing choroidal detachment and exudative retinal detachment on the 1^st^ postoperative day

**Figure 3:**
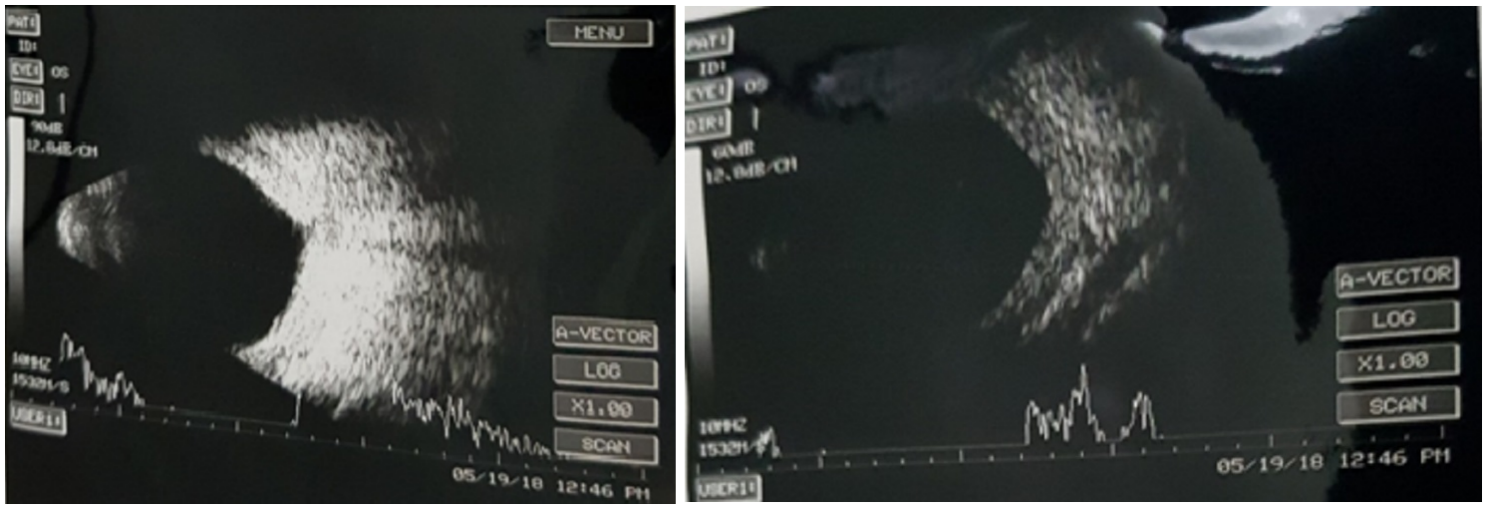
B-scan ultrasonography after 2 weeks in postoperative period showing resolution of choroidal and retinal detachments

On the first postoperative day, the visual acuity was 3/60, the anterior chamber was shallow with choroidal detachment and exudative retinal detachment (Figure 2 (Fig. 2)). For this reason, oral prednisolone 1 mg per kg body weight as a single morning dose was added to the operative medications. The same was gradually tapered off over one month. On the fifth postoperative day, visual acuity improved to 6/60, slit-lamp examination showed a peripheral anterior chamber depth of ½ central corneal thickness. Intraocular pressure was 9 mmHg. Patient was continued on postoperative medications and follow-up visits. Subsequent visit one month postoperatively revealed a best corrected visual acuity of 6/9, a well-formed anterior chamber and normalization of intraocular pressure to 12 mmHg. Fundus examination showed resolution of choroidal and exudative retinal detachment (Figure 3 (Fig. 3)).

## Discussion

Previous studies have equally demonstrated the efficacy of Ahmed valve implant in patients with Sturge-Weber glaucoma [1], [6], [7]. The major surgical concern in our case was the presence of a diffuse choroidal hemangioma which was associated with a higher risk of massive choroidal effusion or haemorrhage and serous retinal detachment during surgery. The best surgical modality for our case was Ahmed glaucoma valve as it has been shown to produce the least postoperative hypotony [7]. This has been attributed to the valve-like mechanism that allows flow of aqueous humour from the eye to the plate. However, patients with SWS receiving Ahmed glaucoma valve implantation still have the potential of developing sight threatening complications of choroidal effusion and haemorrhage when IOP suddenly decreases [7]. Hence, the surgeon needs to anticipate and reduce such a complication by careful attention to maintaining a normal IOP during and after surgery through the use of an anterior chamber maintainer cannula for constant infusion, generous amounts of viscoelastic, and meticulous wound closure. In our index case, the patient had choroidal and retinal detachment with visual deterioration which responded to conservative management. Kaushik et al. reported intractable choroidal effusion in a case which had Ahmed valve implantation following failure of trabeculectomy, which did not respond to initial conservative therapy but responded to oral propranolol, with the effusion resolving over six months [5]. In our case, the patient responded well to initial conservative therapy with aggressive topical and oral steroid therapy. Lavaju et al. reported a case of choroidal effusion following trabeculectomy with 5-FU, which also resolved spontaneously, but nevertheless underlined the importance of a meticulous surgical technique in such cases [6]. Hamush et al. reported Ahmed valve implantation in 11 cases with minimal complications; however, none of the cases had a previous history of filtration surgery as in our case [4].

## Conclusions and recommendations

Ahmed glaucoma valve offers safety and efficacy in controlling glaucoma in paediatric Sturge-Weber syndrome with choroidal haemangioma. However, the increased risk of choroidal and retinal detachment should be kept in mind by surgeons and as such meticulous care should be taken to maintain IOP during surgery and the perioperative period to avoid this devastating complication. These would include steps such as use of an anterior chamber (AC) maintainer, leaving viscoelastics in the AC at the end of surgery, among other methods.

## Notes

### Competing interests

The authors declare that they have no competing interests.

### Acknowledgement

We sincerely thank the management of the Ispahani Islamia Eye Institute and Hospital for providing administrative support to the research.
